# Microfracture for medium size to large knee chondral defects has limited long‐term efficacy: A systematic review

**DOI:** 10.1002/jeo2.70060

**Published:** 2024-10-19

**Authors:** Varun Gopinatth, Garrett R. Jackson, Daniel C. Touhey, Jorge Chahla, Matthew V. Smith, Matthew J. Matava, Robert H. Brophy, Derrick M. Knapik

**Affiliations:** ^1^ Saint Louis University School of Medicine St. Louis Missouri USA; ^2^ Midwest Orthopaedics at Rush University Medical Center Chicago Illinois USA; ^3^ Department of Orthopaedic Surgery Washington University School of Medicine St. Louis Missouri USA

**Keywords:** cartilage, osteoarthritis, chondral, knee, microfracture

## Abstract

**Purpose:**

To evaluate clinical and radiographic outcomes, return to sport, failure rate, operations and complications in patients undergoing microfracture of the knee, including the femoral condyle, tibial plateau, patella and trochlea, at a mean 10‐year or greater follow‐up.

**Methods:**

A literature search was performed by querying SCOPUS, PubMed, Medline and the Cochrane Central Register for Controlled Trials from database inception through May 2023 according to the 2020 Preferred Reporting Items for Systematic Reviews and Meta‐Analyses statement. Inclusion criteria were level I‐IV human studies reporting on outcomes, reoperations and complications following microfracture of the knee at a mean 10‐year or greater follow‐up. Biomechanical and epidemiological studies, including patients undergoing concomitant realignment procedures, and studies with patients under 18 years old were excluded. Data regarding failure, as defined by each study, as well as reoperations were gathered. Study quality was assessed via the Methodological Index for Nonrandomized Studies criteria.

**Results:**

Nine studies from 2003 to 2018, consisting of 727 patients (mean age 38.9 ± 8.1 years; range 25.8–47.6) undergoing microfracture for chondral defects in the knee were identified. Mean follow‐up ranged from 10 to 17 years. Males composed 56.5% of patients, with a mean defect size ranging from 2.3 to 4.01 cm^2^. Based on radiographs at follow‐up, osteoarthritis progression occurred in 40%–48% of patients. Magnetic Resonance Observation of Cartilage Repair Tissue scores were low. Patient‐reported outcome measures showed significant improvement in postoperative scores at final follow‐up. Return‐to‐sport rate ranged from 17.2% to 20%. Longitudinal analysis revealed declining clinical outcomes and return‐to‐sport rates from short‐ and mid‐ to long‐term follow‐up. There was high variability in failure definition and reoperations, with 2.9%–41% of patients requiring total knee arthroplasty.

**Conclusions:**

At a mean 10‐year or greater follow‐up, microfracture for chondral defects of the knee 2–4 cm^2^ in size demonstrated a high rate of osteoarthritis progression with poor healing of the chondral defect and low overall return‐to‐sport rates. Failure and reoperation rates ranged from 2.9% to 41%, with declining outcomes from short‐ and mid‐ to long‐term follow‐up. The advantages of microfracture relating to availability, complexity, and cost should be weighed against concerns about long‐term success, particularly with medium‐size and larger lesions.

**Level of Evidence:**

Level IV systematic review.

**Abbreviations**
ACD: articular cartilage defect
ACI: autologous chondrocyte implantation
ADL: activities of daily living
AP: anterior–posterior
BMI: body mass index
cm: centimeters
CT: computed tomography
F: female
ICRS: international cartilage repair society
IKDC: International Knee Documentation Committee
KOOS: knee osteoarthritis and outcome score
L: left
LFC: lateral femoral condyle
LTP: lateral tibial plateau
M: male
MCID: minimally clinically important difference
MFC: medial femoral condyle
MFX: microfracture
MINORS: methodological index for nonrandomized studies
mKOSS: modified Knee Osteoarthritis Scoring System
mm: millimetres
MOCART: magnetic resonance observation of cartilage repair tissue
MRI: magnetic resonance imaging
MTP: medial tibial plateau
NAS: numeric analogue score
NR: not recorded
OA: osteoarthritis
OAT: osteochondral autograft transfer
OCD: osteochondral defect
PAT: patella
Post‐op: postoperative
Pre‐op: preoperative
PRISMA: preferred reporting items for systematic reviews and meta‐analyses
PROM: patient reported outcome measure
QOL: quality of life
R: right
RCT: randomized controlled trial
SD: standard deviation
TKA: total knee arthroplasty
TRO: trochlea
yrs: years

## INTRODUCTION

Acquired chondral defects of the knee have been reported to be present in up to 61% of patients undergoing knee arthroscopy [13]. The treatment of chondral lesions is dependent on lesion location, size, depth, bony involvement, extremity alignment, meniscal conditions, and the presence of symptomatic defects, with options ranging from benign neglect to arthroscopic debridement to staged chondral restoration [38]. The structural changes imparted in the presence of a chondral defect may impact knee biomechanics, resulting in significant pain and disability [12]. When left untreated, cartilage lesions may progress in size, resulting in the development of premature osteoarthritis (OA), pain, and disability [5]. In patients with smaller chondral defects, generally <2 cm^2^ without bony involvement, microfracture (MFX) has traditionally been utilized to promote chondrocyte and bone marrow stimulation, leading to repair at the site of the defect [7, 23].

However, while MFX remains one of the most popular cartilage procedures for small focal chondral defects of the knee due to its simplicity and cost‐effectiveness [3, 17, 24], the procedure is associated with multiple limitations. Namely, the resultant repair tissue is composed primarily of fibrocartilage which lacks the intrinsic biochemical, viscoelastic, and structural properties present in normal hyaline cartilage [31]. Violation of the subchondral plate has also been shown to increase the risk for subchondral cyst formation while potentially devitalizing the underlying subchondral anatomy [19]. As a result, while short‐term outcomes have reported benefits to MFX with improved knee function and pain [8], investigations assessing mid‐to‐long results have reported less favourable outcomes based on patient‐reported outcomes scores and the rate of conversion to total knee arthroplasty (TKA) [7, 33, 34, 37]. Evaluating the long‐term outcomes of cartilage procedures to develop proper indications for treatment based on patient and defect characteristics is crucial, particularly given the progression in cartilage damage when left untreated [14]. However, controversies continue to exist among surgeons regarding indications for various cartilage‐restoration techniques [4].

The purpose of this investigation was to systematically review the current literature to evaluate long‐term outcomes following MFX for the treatment of focal chondral defects of the knee at a mean 10‐year or greater follow‐up. The focus of this review is on the clinical and radiographic outcomes, return‐to‐sport, failure rate, reoperations and the incidence of any complications related to MFX. The authors hypothesized that MFX would result in a low rate of return to sport, declining clinical outcomes, and a rising rate of failure requiring conversion to TKA at a long‐term follow‐up.

## METHODS

### Search strategy and eligibility criteria

A systematic review was conducted according to the 2020 Preferred Reporting Items for Systematic Reviews and Meta‐Analyses (PRISMA) statement [26]. A literature search identifying studies reporting outcomes of MFX for chondral defects of the knee was conducted on 19 May 2023, using PubMed, MEDLINE, Scopus, the Cochrane Database for Systematic Review and the Cochrane Central Register for Controlled Trials. The search included the following terms combined with Boolean operators: ‘microfracture’, ‘knee’, ‘femoral’, ‘tibial’, ‘condyle’, ‘cartilage’, ‘chondral’, ‘articular’, ‘trochlea’, ‘patella’, ‘Steadman’, ‘outcomes’, ‘results’, ‘drill’, ‘long‐term’, ‘mid‐term’, ‘10‐year’, ‘follow‐up’.

Inclusion criteria consisted of level I‐IV studies written in English or in non‐English language with English translation available, reporting on clinical outcomes following MFX surgery for chondral defects of the knee (femoral condyle, tibial plateau, patella, trochlea) with mean 10‐year or greater follow‐up. Exclusion criteria consisted of non‐English language studies, review articles, technical notes, case reports, editorial commentaries, biomechanical and animal studies, epidemiological and national database studies, studies reporting outcomes at mean or median follow‐up of <10 years, studies reporting on patients undergoing concomitant realignment procedures (e.g., anterior closing wedge osteotomy, distal femoral osteotomy, high tibial osteotomy, tibial tubercle osteotomy), as well as studies including patients under the age of 18. Studies from the same institution with overlapping data were excluded with only the investigation with the latest patient follow‐up included. The performance of concomitant ligamentous (anterior cruciate ligament reconstruction) or meniscal (partial meniscectomy, meniscal repair) surgeries was included in the final analysis.

### Data extraction

Two authors (V. G. and G. R. J.) independently performed an initial title and abstract screening, followed by a full‐text screening to determine whether the inclusion or exclusion criteria were met. A third independent author (D. M. K.) was consulted to discuss any disagreements, during which time no disagreements were encountered. Reference lists from the included studies were reviewed and reconciled to ensure that all relevant studies were identified.

Study characteristics from each article were extracted and included: journal, year published, level of evidence, number of patients, patient demographics (age, sex), mean final follow‐up, knee laterality, body‐mass index, defect size, defect location and MFX surgical technique. Any reported data from radiographs and advanced imaging (magnetic resonance imaging [MRI], computer tomography) were recorded. The definition of surgical, clinical or treatment failure from each study was recorded, as well as the number of patients requiring a subsequent operation. All reported clinical outcomes scores and complications were recorded.

### Risk of bias

The Methodological Index for Non‐Randomized Studies (MINORS) criteria were used to minimize bias and evaluate all included studies by two independent authors (V. G. and G. R. J.). A third independent author (D. M. K.) was consulted for any disagreements and to resolve discrepancies if an assigned score of >2 was encountered. The MINORS is a numerical criteria scale used to evaluate noncomparative, nonrandomized studies, consisting of 12 questions with each question scored with a 0 if not reported, 1 if reported but inadequate or 2 if reported and adequate. The ideal score for noncomparative, nonrandomized studies is 16, while the ideal score for comparative, nonrandomized studies is 24. All studies included in this review were assessed using the MINORS criteria.

### Data analysis

Study characteristics and patient demographic information were gathered and analysed using Microsoft Excel (Version 2206, Microsoft Corporation). When preoperative and postoperative patient‐reported outcome measures were reported, the mean improvement was calculated. Studies reporting clinical outcomes with median and range were converted to mean and standard deviation via the VassarStats calculator from Hozo et al. [15] All forest plots were produced via Open Meta‐Analyst (Version 12.11.14, Tufts University).

## RESULTS

**Figure 1:**
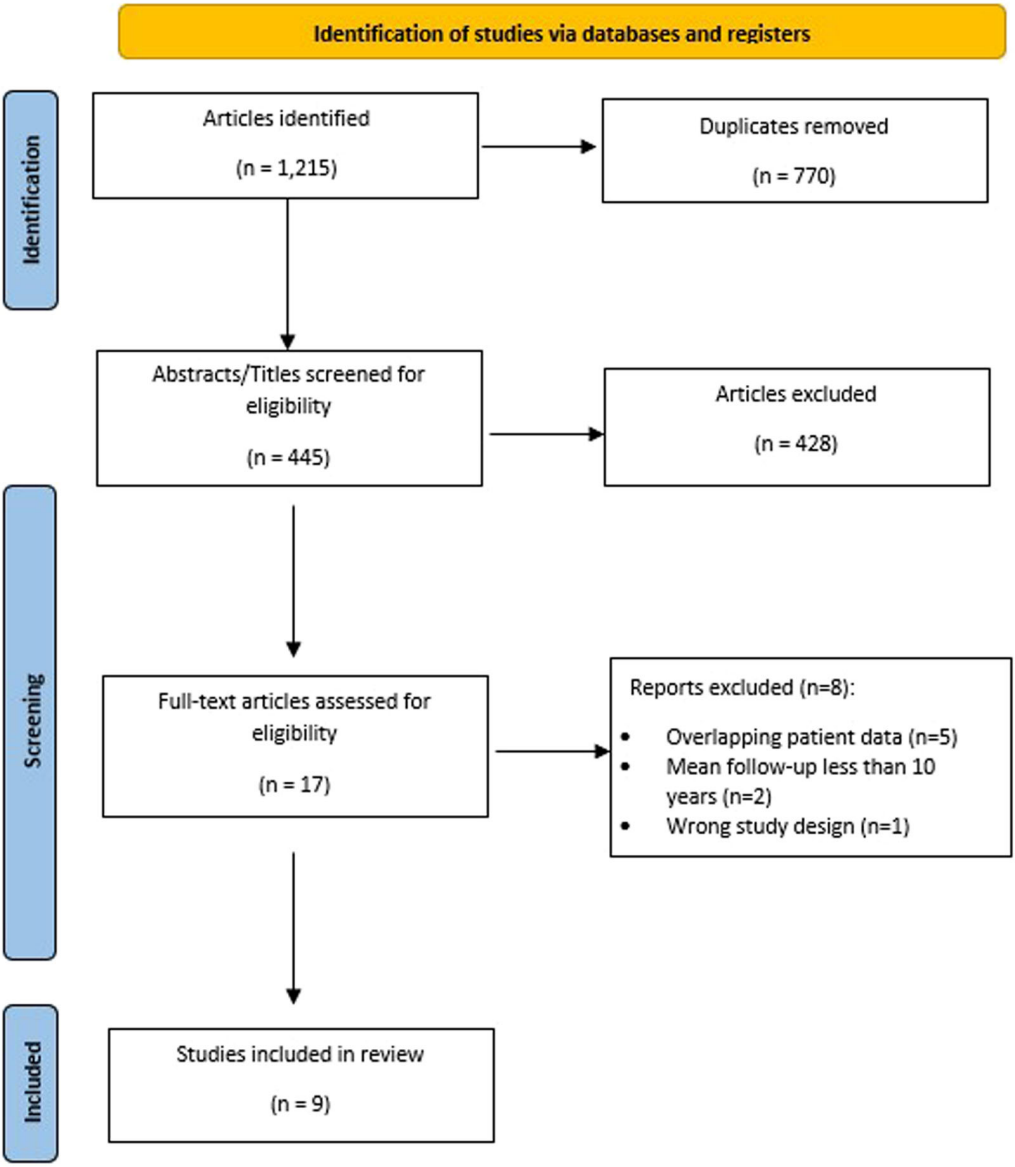
Preferred reporting items for systematic review and meta‐analysis (PRSMA) diagram.

The initial literature search yielded a total of 1215 articles (Figure 1). After duplicates were removed, 445 articles remained and underwent title and abstract screening. Seventeen articles were then selected to undergo full‐text review. Following full‐text review, nine studies were found to meet inclusion criteria and were included in this review.

### Study characteristics

**Figure 2:**
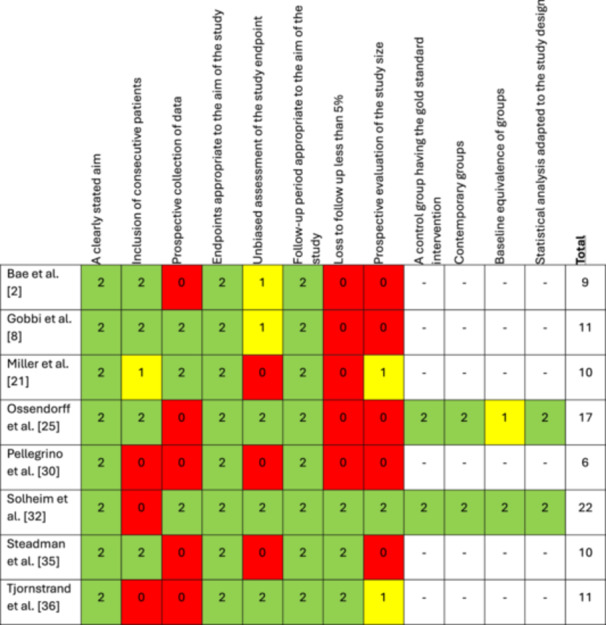
Risk of bias using the methodological index for nonrandomized studies criteria.

Nine studies met inclusion criteria, including one of level I evidence [11], two level III evidence [25, 32] and six‐level IV evidence studies [2, 8, 21, 30, 35, 36] (Table 1). The three comparative studies included a randomized controlled trial (RCT) [11], a comparative study [32] evaluating MFX versus osteochondral autograft transfer (OAT), and a retrospective cohort study comparing MFX versus first‐generation autologous chondrocyte implantation (ACI) [25]. The nine studies included 837 patients. Mean final follow‐up was 12.5 ± 2.8 years (mean range: 10–17 years). The mean overall MINORS score was 12.0 ± 5.1 (range, 6–22). For the six noncomparative, nonrandomized studies, the mean score was 9.5 ± 1.9 (range, 6–11), while the mean score for the two comparative, nonrandomized studies was 19.5 ± 3.5 (range, 17–22) (Figure 2). The randomized controlled trial did not meet criteria to be assessed with the MINORS criteria.

**Table 1:** Overview of included studies.

References	LOE	Intervention	No. of patient's	Mean final follow‐up (range), years
Bae et al. [2]	IV	MFX	124	11.2 (10.0–12.9)
Gobbi et al. [8]	IV	MFX	61	15.1 (10–20)
Gudas et al. [11]	I	MFX vs. OAT	57 (29 MFX, 28 OAT)	10.4 (9–11)
Miller et al. [21]	IV	MFX	350	Min. 10
Ossendorff et al. [25]	III	MFX vs. ACI	44 (22 MFX, 22 ACI)	MFX: 10.2 + −1.7 ACI: 10.6 + −1.1
Pellegrino et al. [30]	IV	MFX	15	11
Solheim et al. [32]	III	MFX vs. OAT	102 (52 MFX, 50 OAT)	16^a^ (15–17)
Steadman et al. [35]	IV	MFX	68	11.3 (7–17)
Tjornstrand et al. [36]	IV	MFX, ACI	16 (6 MFX, 10 ACI)	17 (15–19)

Abbreviations: ACI, autologous chondral implantation; LOE, level of evidence; MFX, microfracture; No, number; OAT, osteochondral allograft transplantation.

^a^ Median value.

### Patient and surgical characteristics

A total of 727 patients (mean age 38.9 ± 8.1; range 25.8–47.6) underwent MFX for chondral defects involving the knee (Table 2). Males made up 56.5% of patients. Mean defect size was reported in seven studies [2, 8, 11, 25, 32, 35, 36], ranging from 2.3 to 4.01 cm^2^. Defect location was reported in six studies, with the most common location being the medial femoral condyle in five studies [8, 11, 25, 32, 36] and the trochlea in one study [35]. The MFX surgical technique typically involved drilling several holes approximately 3–4 mm apart at a depth ranging from 2 to 4 mm deep (Table 2). An awl was most commonly utilized to create the MFX holes [2, 8, 11, 30, 32, 35], while Tjornstrand et al. reported the use of a 2 mm diameter drill [36].

**Table 2:** Study, patient and defect characteristics.

	Bae et al. [2]	Gobbi et al. [8]	Gudas et al. [11] (MFX group)	Miller et al. [21]	Ossendorff et al. [25] (MFX Group)	Pellegrino et al. [30]	Solheim et al. [32] (MFX Group)	Steadman et al. [35]	Tjornstrand et al. [36] (MFX Group)
No. of patients	124	61	29	350	22	15	52	68	6
No. of knees	134	NR	NR	NR	NR	NR	NR	71	NR
Left/right knee	66/68	28/33	NR	NR	13/31	NR	18/34	NR	NR
Sex	20 M/104 F	43 M/18 F	17 M/12 F	229 M/121 F	12 M/10 F	12 M/3 F	30 M/22 F	45 M/23 F	3 M/3 F
Mean age, mean ± SD (range), years	25.8 ± 3.4 (17.5–31.7)	31.4 ± 1.8	24.3 ± 6.8	47.6 (12.1–76.3)	40.5 ± 13.5	44.4	38 ± 11	30.4 (13–45)	38.3 ± 3.67 (34–45)
Mean BMI	NR	NR	Normal	NR	27.3 + − 5.6	NR	NR	NR	NR
Mean preoperative duration of symptoms	NR	NR	21.31 + − 5.7 months	NR	NR	NR	48 (1–360)	166.9 weeks (0.9–839)	NR
Defect size (range)	MFC: 3.5 ± 1.4 cm^2^ (1.0–6.0) MTP: 2.3 ± 1.3 cm^2^ (1.0–3.0)	401.07 ± 26.9 mm^2^	2.77 ± 0.68 cm^2^	NR	2.37 ± 1.6 cm^2^	NR	3.0 cm^2^	277.4 mm^2^ (20–1000)	236.7 ± 92.01 mm^2^ (150–400)
Defect locations	NR		MFC/LFC	NR		NR	MFC		MFC
MFC	‐	39	‐	‐	12	‐	‐	21	6
LFC	‐	20	‐	‐	1	‐	‐	14	‐
MTP	‐	2	‐	‐	‐	‐	‐	1	‐
LTP	‐	7	‐	‐	‐	‐	‐	7	‐
PAT	‐	6	‐	‐	5	‐	‐	16	‐
TRO	‐	9	‐	‐	4	‐	‐	29	‐
MFX hole drilling	Awl	Awl	Awl	NR	NR	Awl	Awl	Awl	Drill
MFX hole spacing	Several holes, 3–4 mm apart	3–4 mm apart	3–4 mm apart	NR	NR	3–4 mm apart	3–4 mm apart	3–4 mm apart	6 mm apart
MFX hole depth	NR	2–4 mm deep	3–4 mm deep	NR	NR	NR	NR	3–4 mm deep	2 mm depth

Abbreviations: BMI, body mass index; cm, centimeters; F, female; L, left; LFC, lateral femoral condyle; LTP, lateral tibial plateau; M, male; MFC, medial femoral condyle; mm, millimetres; MTP, medial tibial plateau; NR, not recorded; PAT, patella; R, right; TRO, trochlea.

### Postoperative rehabilitation

During the first 6–8 weeks, patients were generally nonweightbearing followed by gradual progression with the use of crutches until being weight‐bearing as tolerated [8, 32, 35], typically around 8 weeks following surgery [2, 8, 11, 35]. A continuous passive motion device was routinely recommended for a total of 6–8 h daily during the initial postoperative recovery phase [2, 8, 11, 32, 35]. Steadman et al. also reported the use of cold therapy for the first 1–7 days [35]. Two studies reported on the initiation of physiotherapy following surgery [32, 36]. Bae et al. allowed patients to begin linear running and jumping at 4 months [2]. Return to sport was allowed at 4–6 months in two studies [11, 35] and between 6–8 months in one study [2].

### Imaging outcomes

Radiographic outcomes were reported in four studies [2, 8, 11, 36]. Bae et al. observed a nonsignificant decrease from preoperative to final follow‐up mechanical axis percentage (the percentage by which the mechanical axis bisects the total tibial width). While no significant change in tibiofemoral joint space width on anterior–posterior radiographs was appreciated, a significant 0.7 mm increase in joint space width on lateral radiographs from preoperative to final follow‐up measurements was reported (*p* = .002) [2]. Gobbi et al. reported the development of degenerative changes in 40% (*n* = 24/61) of knees, with greater degenerative development observed in patients greater than 31 years old and those with larger or multiple chondral defects [8]. OA was present in 48% of patients in the investigation by Gudas et al. based on Kellgren–Lawrence grading when compared with preoperative gradings [11]. Meanwhile, Tjornstrand et al. reported on the development of radiographic OA in all six knees treated with MFX [36].

Outcomes based on MRI were reported in two studies [11, 25]. In both studies, cartilage was evaluated using the Magnetic Resonance Observation of Cartilage Repair Tissue (MOCART) score. Gudas et al. reported the percentage of patients achieving MOCART criteria at 10‐year follow‐up, with 35% possessing complete defect repair and filling, 35% with complete integration to the border zone, 76% with intact reapair tissue surface, 38% with homogenous repair tissue structure, 38% with intact subchondral lamina, 41% with intact subchondral bone, 0% with adhesions, and 69% with effusions [11]. Ossendorff et al. reported a mean postoperative MOCART score of 54.1 ± 2.8 and a mean modified Knee Osteoarthritis Scoring System score of 11.9 ± 2.0 [25].

### Clinical outcomes

**Figure 3:**
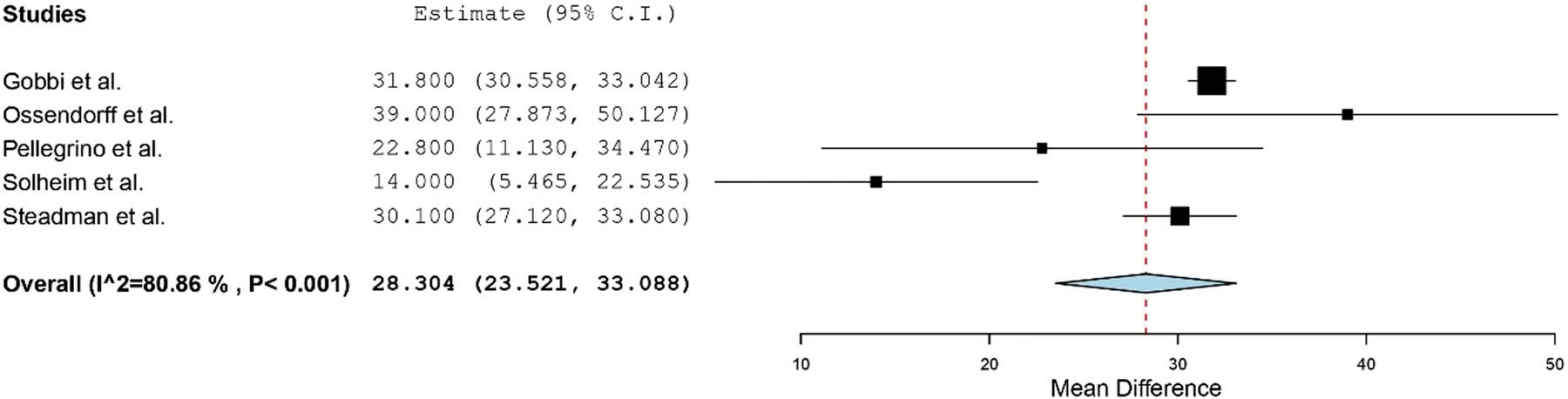
Forest plot showing mean improvement in Lysholm score from preoperative to postoperative levels evaluated at minimum 10‐year follow‐up.

**Figure 4:**
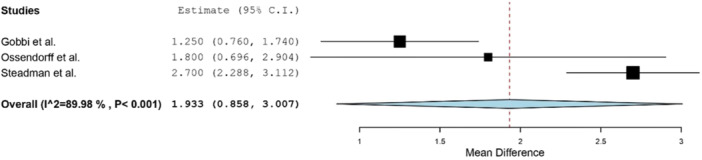
Forest plot showing mean improvement in Tegner score from preoperative to postoperative levels evaluated at minimum 10‐year follow‐up.

Clinical outcomes following MFX were reported in eight studies (Table 3) [2, 8, 11, 25, 30, 32, 35, 36]. The most commonly reported patient‐reported outcomes measures (PROMs) were Lysholm (five studies) [8, 25, 30, 32, 35], Tegner activity (four studies) [8, 11, 25, 35] and International Knee Documentation Committee (IKDC) score (3 studies) [8, 25, 30]. At minimum 10‐year follow‐up, Lysholm scores were significantly improved relative to preoperative levels (Figure 3). Similarly, Tegner scores at final follow‐up were improved when compared to preoperative levels (Figure 4). Postoperative IKDC subjective scores, reported in three studies [8, 25, 30], ranged from 68.7 to 72. Miller et al. conducted a longitudinal data‐analysis in patients undergoing MFX out to 12‐year follow‐up and observed a stable Tegner score over time, with a slowly declining Lysholm score after 2 years [21]. Decreases in pain on subjective outcome rating were reported in two studies [25, 35]. Specifically, Ossendorff et al. [25] reported a mean decrease of 5 points on a numeric analogue scale, while Steadman et al. [35] reported a mean decrease (improvement) of 1.5 points on their subjective questionnaire from preoperative to postoperative levels.

**Table 3:** Clinical outcomes of microfracture at minimum 10‐year follow‐up.

	Lysholm	IKDC subjective	IKDC objective	KOOS	Tegner	Pain	Range of motion	Return to sport	Osteoarthritis progression
Bae et al. [2]	NR	NR	NR	NR	NR	NR	Pre‐op: 134.6 ± 8.9. Post‐op: 137.8 ± 10.3	NR	NR
Gobbi et al. [8]	Pre‐op: 45.4 ± 3.5 Post‐op: 77.2 ± 3.5	Pre‐op: 46.7 ± 2.9 Post‐op: 71.5 ± 4.0	Pre‐op: 0 A, 6 B, 35 C, 20 D Post‐op: 10 A 23 B, 19 C, 9 D.	Post‐op, Pain: 89.6 ± 4.3 Symptoms: 85.6 ± 4.0 ADL: 92.0 ± 3.1 Sports: 74.5 ± 5.9 QOL: 82.2 ± 5.2	Pre‐op^a^: 3 (2–5) Post‐op^a^: 4 (2–8)	Post‐op: 1.6 ± 0.5	Pre‐op: 132.2 ± 3.5 Post‐op: 132.2 ± 1.0	20% return to sport	Post‐op Ahlback: 13 grade 1 six grade 2 four grade 3 one grade 4
Gudas et al. [11]	NR	NR	NR	NR	ACD Post‐op: 6.2 ± 0.4 OCD Post‐op: 6.1 ± 0.7	NR	NR	Same level ACD: 5/10 Same level OCD: 0/5	Kellgren Lawrence Grade 1: 14/29 patients
Ossendorff et al. [25]	Pre‐op: 43 ± 22 Post‐op: 82 ± 15	Post‐op: 72 ± 20	NR	Post‐op, symptoms: 79 ± 18, Pain: 80 ± 21, ADL: 87 ± 17, Sports: 74 ± 26, QOL: 64 ± 26	Pre‐op: 2.8 ± 2.4 Post‐op: 4.6 ± 1.3	NAS Pre‐op: 7.1 ± 2.7 Post‐op: 2.1 ± 2.0	NR	NR	KOOS post‐op: 11.9 ± 2.9
Pellegrino et al. [30]	Pre‐op: 59.33 ± 18.2 Post‐op: 82.13 ± 14.16	Pre‐op: 45.13 ± 17.07 Post‐op: 68.66 ± 21.47	NR	NR	NR	NR	NR	NR	NR
Solheim et al. [32]	Pre‐op: 48 ± 19, Post‐op: 62 ± 25.	NR	NR	NR	NR	NR	NR	NR	NR
Steadman et al. [35]	Post‐op: 88.9 ± 7.3 Chang: 30.1 ± 12.3 Poor Lysholm (<65): 13/20 (65%), Good Lysholm (>80): 4/20	NR	NR	NR	Post‐op: 5.8 ± 1.5 Change: 2.7 ± 1.7	Post‐op: 1.9 ± 0.5 Change: −1.5 ± 0.9	NR	NR	NR
Tjornstrand et al. [36]	NR	NR	NR	NR	NR	NR	NR	NR	Radiographic osteoarthritis progression (6)

Abbreviations: ADL, activities of daily living; ACD, articular cartilage defect; NR, not recorded; OCD, osteochondral defect; post‐op, postoperative; pre‐op, preoperative; QOL, quality of life.

^a^ Median values listed.

### Return to sport

The return‐to‐sport rate was reported in two studies [8, 11], ranging from 17.2% to 20%. Gobbi et al. reported that 60% of patients returned to sport at a 2‐year follow‐up, declining to 20% at a mean final follow‐up of 15.1 years [8]. Gudas et al. reported an overall return‐to‐sport rate of 17.2% at a mean 10.4‐year follow‐up [11].

### Failures, reoperations and complications

The incidence of MFX failure and reoperations was reported in seven studies [2, 8, 11, 25, 32, 35, 36], with 2.9%–41% of patients requiring subsequent surgery (Table 4). Bae et al. reported that 41% (*n* = 51/124) of patients required TKA at a mean of 6.8 years [2]. Meanwhile, six patients were reported as having sustained clinical failure based on follow‐up pain scores being worse when compared to preoperative scores. The survival rate of MFX dropped from 88.8% at 5 years to 67.9% at 10 years and 45.6% at 12 years [2]. Solheim et al. observed that 15% (*n* = 5/52) of patients required TKA at a mean of 12 years [32]. Gobbi et al. reported that 11% (*n* = 7/67) of patients underwent reoperation due to reinjury or persistent pain [8], with 38% (*n* = 11/29) of patients requiring reoperation due to persistent symptoms as reported by Gudas et al. [11] Tjornstrand et al. observed that 100% (*n* = 6/6) of patients were classified as having sustained clinical failure due to progression of OA, including one patient undergoing unicompartmental knee arthroplasty [36]. Steadman et al. reported failures in two patients (2.9%, *n* = 2/68), with one patient sustaining a fall requiring a Fulkerson tibial tubercle osteotomy and another requiring a revision MFX procedure [35]. Subsequent procedures were required in 23% (*n* = 5/22) of patients by Ossendorff et al. including synovial‐/plica‐ resection (*n* = 2), abrasion (*n* = 2), and an undefined meniscal procedure (*n* = 1) [25].

**Table 4:** Failures, reoperations and complications of microfracture at minimum 10‐year follow‐up.

	Failure definition	No. of failures	No. of reoperations	Complications
Bae et al. [2]	Need for TKA during follow‐up period Clinical failure: Pain score lower than preoperative score Need for total knee arthroplasty Clinical Failure = Final Follow‐up pain score lower than preoperative pain score	51 (Conversion to TKA) six (Clinical Failure) 5‐year survival: 88.8% 10‐year survival: 67.9% 12‐year survival: 45.6%	*n* = 51/124 (41%) TKA (*n* = 51)	‐‐
Gobbi et al. [8]	Underwent reoperation due to reinjury or persistent pain Need for reoperation	*n* = 7/67 (11%)	*n* = 7/67 (11%)	Superficial infection (*n* = 1)
Gudas et al. [11]	Needed a reoperation within 10 years due to symptoms of a primary defect Need for reoperation	*n* = 11/29 (38%)	*n* = 11/29 (38%)	‐‐
Ossendorff et al. [25]	‐‐	‐‐	*n* = 5/22 (23%) Synovial‐/Plica‐resection (*n* = 2); Meniscus (*n* = 1); Abrasion (*n* = 2)	‐‐
Solheim et al. [32]	‐‐	‐‐	*n* = 5/52 (10%) TKA (*n* = 5)	None
Steadman et al. [35]	Not defined	*n* = 2/68 (2.9%)	*n* = 2/68 (2.9%) Fulkerson osteotomy (n = 1); Revision MFX (*n* = 1)	Mild pain (*n* = 38); Moderate pain (*n* = 10)
Tjornstrand et al. [36]	Progression of radiographic osteoarthritis	*n* = 6/6 (100%)	UKA (*n* = 1)	‐‐

Abbreviation: TKA, total knee arthroplasty; UKA, unicompartmental knee arthroplasty.

Complications were reported in two studies [8, 35]. A superficial infection was reported by Gobbi et al.,^17^ while Steadman et al. reported that 38 patients experienced mild pain and 10 patients experienced moderate pain following MFX [35].

### Long‐term efficacy of MFX compared to chondral restoration

Three studies compared the long‐term results of MFX to chondral restoration procedures, including one RCT [11] and two retrospective cohort studies [25, 32]. The RCT by Gudas et al. compared patients undergoing MFX versus OAT [11]. At a mean 10.4‐year follow‐up, the authors reported significant improvements following OAT when compared to MFX based on the International Cartilage Repair Society score, Tegner activity scores and a higher rate of return to preinjury sports activities (OAT: 10/29, MFX: 5/29). Patients undergoing MFX were also noted to experience a significantly higher failure rate (14% OAT, 38% MFX, *p* < .05). OAT was also reported to yield superior outcomes based on MOCART criteria, including a greater proportion of knees achieving complete filling of the defect, complete integration of the border zone, intact surface area, intact subchondral lamina, and intact subchondral bone. Meanwhile, in the comparative study by Solheim et al., patients undergoing OAT reported significantly higher Lysholm scores compared to MFX at 6 months, 12 months, 5 years, and at the 10‐year follow‐up, with no differences reported at the 15‐ to 18‐year follow‐up [32]. There was no significant difference in conversion to TKA between groups (MFX: 10%, OAT: 16%). The cohort study by Ossendorff et al. comparing MFX to first‐generation ACI observed that patients undergoing MFX underwent significantly fewer reoperations (23 ACI, five MFX) and exhibited significantly higher postoperative Lysholm and KOOS sport and quality of life scores compared to ACI [25]. The mean defect size for patients in the MFX group was 2.37 ± 1.6 cm^2^ compared to 4.74 ± 2.3 cm^2^ in the ACI group.

## DISCUSSION

The primary findings of this systematic review were that patients undergoing MFX for medium‐size to large full‐thickness chondral defects, most commonly of the femoral condyles, had improved PROMS compared to preoperative values despite progressive degenerative changes that were noted in 40%–100% of patients at a mean follow‐up of 10 years or greater. The return‐to‐sport rate reported in two studies ranged from 17.2%–20%, with failure rates following ranging from 2.9% to 41%. As the mean chondral defect size ranged from 2.4–4.0 cm^2^, this systematic review corroborates previous recommendations against MFX for larger lesions due to poor long‐term outcomes, which may be best treated using ACI or osteochondral allograft [3, 6, 30].

Clinical outcomes following MFX were most commonly assessed with the Lysholm, Tegner and IKDC scoring scale. Five studies reporting on Lysholm scores reported statistically significant postoperative improvements at a mean 10‐year or greater follow‐up [8, 25, 30, 32, 35]. Similar results were seen in studies that reported preoperative and postoperative Tegner scores. Our study found a mean improvement in Lysholm scores ranging from 14–31.8, similar to the long‐term improvement reported in studies evaluating outcomes following OAT (21.1) [28] and ACI (16.5) [27], but lower than osteochondral allograft transfer (53) [1]. Similarly, the mean long‐term improvement in Tegner scores ranged from 1.25–2.70, similar to outcomes reported following OAT (0.76) [28] and ACI (1.1) [27]. Furthermore, outcome scores were found to progressively decline with longer follow‐up, which is a known limitation of the MFX technique [8, 21, 22]. Solheim et al. reported a preoperative mean Lysholm score of 48, peaking at 72 at a 6‐month follow‐up and diminishing to 62 by 15‐ to 18‐year follow‐up [32]. Similar results were observed by Gobbi et al., who reported a preoperative mean Lysholm score of 45.4, peaking at 90.4 at a 2‐year follow‐up and declining to 77.2 at final follow‐up [8]. The longitudinal data analysis performed by Miller et al. generated a profile plot demonstrating a decline in Lysholm scores over time up to 12 years [21]. This decline was not observed in the Tegner scores. Gobbi et al. also observed only a 1‐point drop in Tegner score between a 2‐year and 15‐year follow‐up. When successful, filling of the chondral defect utilizing the MFX technique improves joint congruency [20]. However, the questionable mechanical integrity of fibrocartilage may lead to degeneration of the repair tissue and worsening clinical outcomes over time. Therefore, cell‐based therapy may be a more suitable option with greater long‐term durability due to the production of cartilage that is more stable and allows for better integration [18].

The reported return‐to‐sport rate following MFX ranged from 17.2% to 20%. Both studies reporting on return to preinjury levels of sports observed a declining rate over the course of a long‐term follow‐up. Specifically, Gobbi et al. observed a drop in return to sport from 60% to 20% from 2‐ to 15‐year follow‐up [8]. Similarly, Gudas et al. observed a decline from 51.7% to 17.2% at a 10‐year follow‐up [11]. The observed long‐term return to sport for patients undergoing MFX was lower compared to the reported 85% rate reported following an OAT [28]. Therefore, for younger, athletic patients seeking a return to sports or an active lifestyle, surgeons must consider the long‐term implications when treating patients with a chondral defect of the knee using the MFX procedure. The observed decline in sport participation may warrant consideration of other surgical options to restore the damaged chondral surface based on lesion size and depth [6, 28].

The reported definition of failure following MFX was highly heterogeneous, including conversion to TKA, reoperations, clinical failure (pain score at follow‐up lower than preoperative score) and treatment failure. A reoperation rate ranging from 2.9% to 41% was appreciated, with survival rates declining with increasing follow‐up. Specifically, Bae et al. observed a declining survival rate from 88.8% at 5 years to 45.6% by 12 years [2]. High failure rates and declining survival were most commonly due to OA progression, generally confirmed radiographically. Gobbi et al. [8] reported degenerative changes occurring in 40% of knees, while Gudas et al. [11] observed Grade I Kellgren–Lawrence OA development in 48% (*n* = 14/29) of patients compared to no arthritic changes in any patients preoperatively. Long‐term MFX failure and reoperation rates were similar to those reported at long‐term follow‐up for other cartilage procedures including OAT (mean follow‐up: 10.2 years; failure rate: 28%, reoperation rate 19%) [28], osteochondral allograft transfer (mean follow‐up: 12.3 years; failure rate: 25%, reoperation rate: 36%) [1] and ACI (mean follow‐up: 11.4 years; failure rate: 18%, reoperation rate: 37%) [27].

The long‐term outcomes following MFX compared to chondral restoration procedures remain largely unknown. Meta‐analyses evaluating clinical outcomes have demonstrated superior results following OAT and ACI compared to MFX at short‐ to mid‐term follow‐up. Specifically, this includes a higher quality of repaired cartilage, lower failure rates, lower re‐operation rates and higher return‐to‐sport rates [9, 29]. Moreover, a recent systematic review found it difficult to assess the efficacy of cartilage restorative procedures with respect to minimal clinically important difference due to variable follow‐up and lack of data for certain procedures [16]. While OAT was reported to yield improved outcomes at long‐term follow‐up compared to MFX, comparative results following ACI require further investigation utilizing newer generation ACI techniques. Long‐term prospective studies are necessary to compare the efficacy of cartilage‐restoration techniques.

### Limitations

This investigation is not without limitations. Study quality, based on level of evidence, as well as the degree to which defect, surgical technique and outcome details reported were highly heterogeneous, leading to a likely selection bias toward specific studies providing comprehensive data. The level of evidence of included studies varied, ranging from I to IV. Included studies were limited primarily to investigations examining outcomes for medium‐ to large‐chondral defects, making it difficult to determine long‐term outcomes following MFX for smaller, contained lesions, which have been shown to be ideal for MFX treatment [10]. The lack of a control group among many of the studies makes it difficult to decipher the true effect of treatment on outcomes given the natural decline in sports participation and OA progression with age. Due to heterogeneity in studies reporting drop‐out rates, and failure definitions, pooling of failure and reoperation rates was avoided. This limited the ability of the authors to perform any meaningful statistical analyses or a meta‐analysis to synthesize data based on the current literature. Only nine studies met the inclusion criteria, resulting in a small patient population and a high variability in failure and reoperation rates. The search strategy and inclusion criteria were done in accordance with PRISMA guidelines, but it is possible that included studies may have been left out of the literature search. One of the significant limitations of the current review is that the lesions in the included studies were on the larger end of those typically treated with MFX (i.e., 2.3–4 cm compared to <2 cm). This could be an important confounder as the limitations of MFX are likely inversely related to lesion size.

## CONCLUSION

At a mean 10‐year or greater follow‐up, MFX for chondral defects of the knee 2–4 cm^2^ in size demonstrated a high rate of OA progression with poor healing of the chondral defect, and low overall return‐to‐sport rates. Failure and reoperation rates ranged from 2.9% to 41%, with declining outcomes from short‐ and mid‐ to long‐term follow‐up. The advantages of MFX relating to availability, complexity, and cost should be weighed against concerns about long‐term success, particularly with medium‐size and larger lesions.

## AUTHOR CONTRIBUTIONS

**Varun Gopinatth**: Study conceptualization; data curation; formal analysis; investigation; methodology; project administration; study validation; data visualization and presentation; manuscript draft preparation; manuscript review and editing. **Garrett R. Jackson**: Data curation; formal analysis; investigation; methodology; data visualization; manuscript draft preparation. **Daniel C. Touhey**: Data curation; formal analysis; investigation; methodology; data visualization; manuscript draft preparation. **Jorge Chahla**: Methodology; resources; supervision; validation; manuscript draft preparation; manuscript review and editing. **Matthew V. Smith**: Methodology; resources; supervision; validation; manuscript draft preparation; manuscript review and editing. **Matthew J. Matava**: Methodology; resources; supervision; validation; manuscript draft preparation; manuscript review and editing. **Robert H. Brophy**: Methodology; resources; supervision; validation; manuscript draft preparation; manuscript review and editing. **Derrick M. Knapik**: Study conceptualization; data curation; investigation; methodology; project administration; resources; supervision; validation; manuscript draft preparation; manuscript review and editing.

## CONFLICTS OF INTEREST STATEMENT

J. C. has received consulting fees from Arthrex, CONMED Linvatec Corporation, Ossur, Smith & Nephew, Vericel, Stryker Corporation and DePuy Synthes Products; support for education from Arthrex, Medwest Associates and Smith & Nephew; speaking fees from Linvatec, Arthrex and Smith & Nephew; hospitality payments from Medical Device Business Services, Medwest Associates, Smith & Nephew, Linvatec and Stryker and a grant from Arthrex. M. V. S. has received speaking and faculty, education and hospitality payments from Arthrex and education and hospitality payments from Elite Orthopaedics. M. J. M. has received consulting, faculty & speaking and hospitality payments from Arthrex; education payments from Elite Orthopaedics; consulting and hospitality payments from Heron Therapeutics and consulting and hospitality payments from Pacira Pharmaceuticals. R. H. B. has received support for education from Elite Orthopaedics. D. M. K. has received support for education from Smith & Nephew, Elite Orthopaedics and Medwest Associates; hospitality payments from Arthrex, Encore Medical, Stryker and Smith & Nephew; honoraria from Encore Medical and a grant from Arthrex. The remaining authors declare no conflict of interest.

## ETHICS STATEMENT

The authors have nothing to report.

## Data Availability

Data generated and analysed in this study is within the published manuscript.
